# Post-reproductive lifespan in wild mountain gorillas

**DOI:** 10.1073/pnas.2510998122

**Published:** 2025-10-13

**Authors:** Nikolaos Smit, Martha M. Robbins

**Affiliations:** ^a^Department of Primate Behavior and Evolution, Max Planck Institute for Evolutionary Anthropology, Leipzig 04103, Germany; ^b^Department of Biology, University of Turku, Turku 20014, Finland

**Keywords:** postreproductive lifespan, reproductive senescence, menopause, gorillas

## Abstract

Animals can typically maximize their fitness by reproducing throughout adulthood. Yet, in a handful of species, females cease reproduction long before death, highlighting an apparent evolutionary paradox. We used over three decades of life-history and behavioral data to examine the prevalence of postreproductive lifespan in wild mountain gorillas (*Gorilla beringei beringei*). Almost one third of females in our study population (7/25) have been “postreproductive” according to a commonly used criterion and have lived more than a decade past their age of last reproduction, representing at least a fourth of their adult lifespan. Additionally, using conservative estimates of female ages, we found a significant post-reproductive representation (a common population-level measure of post-reproductive lifespan) equal to 0.10. Our results add to observations of postreproductive lifespan in chimpanzees and humans and thus, they represent a critical addition to our understanding of hominid life-history evolution.

Postreproductive lifespan constitutes an apparent evolutionary paradox, as it is unclear whether, and under what conditions, reproductive cessation can confer fitness benefits. Since female mammals can increase their fitness by reproducing until the end of their lives, why would they cease reproduction long before it? Aside from humans, postreproductive lifespan has been documented only in a handful of long-lived mammals, mostly toothed whales (1–3), making its study difficult and reinforcing its apparent evolutionary paradoxical nature. To decipher the evolution of postreproductive lifespan, observations of closely related species, including those closely related to humans, are critical.

A recent study documented postreproductive lifespan (specifically, menopause) in the population of wild chimpanzees (*Pan troglodytes*) exhibiting the longest life expectancy [Ngogo; (4)]. Gorillas, close relatives to chimpanzees and humans, exhibit similar average life expectancy at birth to chimpanzees [gorillas, mean ± SD: 17.9 ± 2.9 y; chimpanzees: 19.7 ± 10.7; (5, 6)], providing the opportunity to comparatively test the theories regarding the evolution of postreproductive lifespan. Fertility in female gorillas declines with age (7). More than half of captive western gorilla (*Gorilla gorilla gorilla*) females older than 30 y do not exhibit estrus cycles or they exhibit variable hormonal patterns suggesting perimenopause [i.e., insufficient increases in progestogen levels during the luteal phase; (8)], similar to a 38-y-old wild female mountain gorilla [*Gorilla beringei beringei*; (9)]. Yet, previous studies have not identified extended postreproductive lifespan in gorillas [1 to 3% of total female lifespan of Virunga mountain gorillas (7)]. Similar to chimpanzees, different gorilla populations may exhibit variation in life expectancy and postreproductive lifespan (4, 8). Here, we investigate postreproductive lifespan in the other population of mountain gorillas, that in Bwindi Impenetrable National Park, Uganda.

## Results

**Fig. 1.**
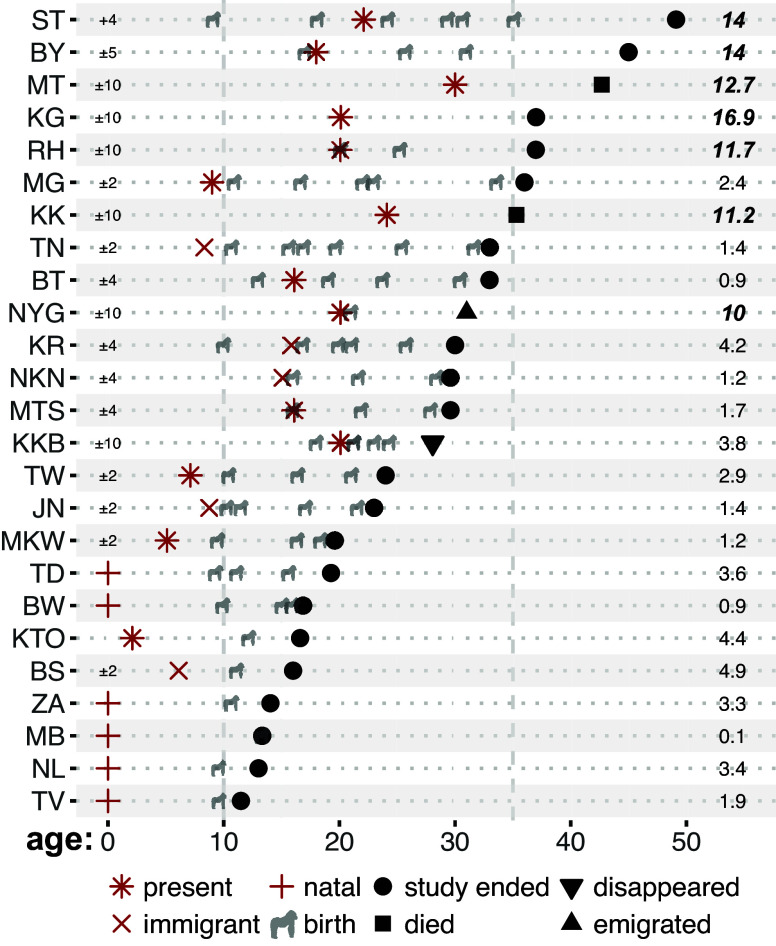
Years since last reproduction (*Right*; values over 7.7 y are in bold to denote postreproductive females) and errors of birth date estimation in years (*Left*, next to female identities; exact birth dates were known for six females). Female ages when entering to the study are marked by orange symbols: 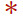 for females present in the study groups upon habituation, 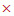 for female immigrants, and 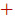 for females born during the study. Ages when giving birth are shown by gray gorilla silhouette icons obtained from https://phylopic.org. Consecutive births are occasionally close in time when infants died soon after birth and the mother conceived shortly again; the second birth/point of KKB is darker because she gave birth to twins. Ages when exiting the study are shown by black symbols: ∙ for females still in the groups at the end of the study (January 2025), ▪ for females who died, ▾ for females who disappeared (unknown if died or emigrated), and ▴ for females who emigrated to nonhabituated groups.

**Fig. 2.**
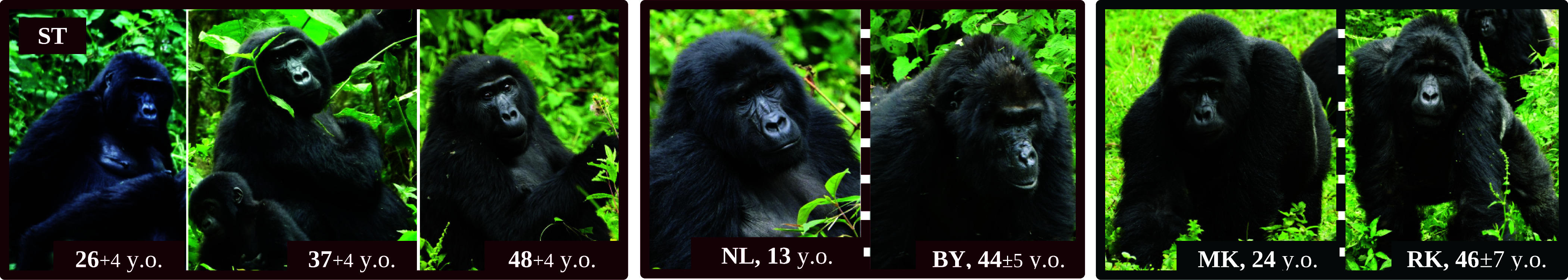
Mountain gorillas. (*A*) The oldest female in the study population exhibited a good body condition even at 48 y old (minimum possible age). (*B*) A young and an old female. (*C*) A prime aged and an old male.

We used life-history and behavioral data (10) from four wild groups of mountain gorillas in Bwindi Impenetrable National Park, Uganda, to calculate postreproductive lifespan of 25 adult females. According to a commonly used definition, “postreproductive females” are those who live past the age of their last reproduction for longer than the mean plus two SD of successful interbirth intervals (2). We calculated this value as 7.7 y [5.1 + (2 × 1.3)] in our study population, suggesting that seven out of the 25 study females qualified as postreproductive. Six of these seven females have been conservatively estimated (based on the ages of genetically identified offspring, body condition and hair loss) to be older than 35 y old, which is the maximum age of observed reproduction (Figs. 1 and 2). All the seven postreproductive females exhibited a postreproductive lifespan of at least 10 y (Fig. 1), minimizing the possibility to be “mistakenly” classified as postreproductive. These females were not observed mating for an average of 7.4 ± 5.8 y before they exit the study (average number of days with behavioral observations of their groups over this period: 1,006 ± 762), in line with observations of declining mating activity with age (7).

We also calculated postreproductive representation (PrR), a common population-level measure of postreproductive lifespan which incorporates both the probability of female survival to reproductive cessation and life expectancy beyond reproductive cessation (11). PrR was equal to 0.10 and significantly greater than zero (*P*-value < 0.001), providing statistical support for the presence of postreproductive lifespan in mountain gorillas. To test the influence of age estimate errors on PrR value and significance, we ran 1,000 simulations incorporating age uncertainty. The mean PrR value was 0.094 ± 0.023 (±SD) and all corresponding *P*-values were <0.05, indicating that our findings are robust to variation in age estimation.

## Discussion

Our study shows that wild Bwindi mountain gorillas can exhibit long postreproductive lifespans. Given that female gorillas rarely reach 50 y of age in the wild (6), the 10 postreproductive years lived by one third of the study females represents at least 25% of their adult lifespan (adults: >10 y old). More generally, the standardized population measure of PrR suggested that females spend 10% of their adult lifespan as postreproductive. Importantly, neither of the two methods we used to derive postreproductive lifespan can distinguish menopause from other causes of sterility, such as an increased fetal loss probability in old females. Nevertheless, the extensive duration of postreproductive lifespan, the reduced or lack of mating activity, and previous endocrine analyses of old females (8, 9) suggest that menopause is a highly plausible cause for the reproductive patterns we observed. The selective pressure(s) which might have favored the evolution of this trait in gorillas remain unclear. Below we discuss the prominent hypotheses for the evolution of postreproductive lifespan across species, and their predictive power in gorillas.

The “reproductive conflict hypothesis,” posits that old females cease reproduction to avoid competition for limited reproductive opportunities with young (related) individuals (12); e.g., their daughters or the mates of their sons]. Female gorillas disperse from their natal groups and often disperse again from groups where they have reproduced (13), meaning that they have low relatedness to their groupmates. Hence, the benefits of reproduction for female gorillas at an old age may be greater than that for chimpanzees or humans, where female local relatedness increases with age and females reproduce simultaneously with their offspring (12, 14).

Another relevant set of hypotheses, also relatively unlikely to apply to gorillas, posit that intergenerational help, and its positive influence in grandoffspring fitness, may drive the evolution of postreproductive lifespan through two not mutually exclusive evolutionary pathways [see also “grandmother hypothesis”; (1)]: by selecting for longer female lifespan to allow females overlap with grandoffspring and help them increase their fitness (e.g., by offering their ecological knowledge, or by defending them), and/or by selecting against longer reproductive female lifespan when females gain higher (indirect) fitness by helping their grandoffspring to survive or reproduce, rather than reproducing themselves (1, 3). In contrast to some toothed whales and humans, such intergenerational help is less probable in gorillas as both females and males typically disperse from their natal groups to reproduce (13), limiting coresidence of kin and opportunities for females to help in grandoffspring survival and more generally in offspring reproduction.

The associated “mother hypothesis” (15) might have greater predictive power in gorillas. This hypothesis posits that old females cease reproduction to minimize energy expenditure or other reproductive costs, and maximize investment to existing offspring and their fitness. Consistent with this hypothesis, maternal presence, care, and support is critical even for adults in gorillas and other hominids (16). Female gorillas who cease reproduction can show a greater body condition than old females who reproduce (pers. obs. MMR; Fig. 2), suggesting that they might have a greater ability to support existing offspring; but future research may clarify or reject this hypothesis.

A final hypothesis posits that postreproductive lifespan is a nonadaptive by-product of life-history patterns. Given that many wild animals die from predation, disease, or starvation, genes whose deleterious effects appear only in advanced ages, may not be purged (15). When “favorable” conditions allow individuals to survive at these ages, deleterious effects that prevent reproduction may appear (4, 11). Accordingly, greater food abundance and potentially lower predation pressure in comparison to the evolutionary history of chimpanzees, may allow Ngogo chimpanzees to live longer and exhibit menopause (4). Similarly, Bwindi gorillas currently do not face any predation risk from leopards, their main potential nonhuman predators, and habituated individuals occasionally receive veterinary care due to their endangered status, potentially enabling longer lives and the documentation postreproductive lifespan. However, the seven identified postreproductive females have never received veterinary care (pers. obs. MMR), and Bwindi gorillas do not live much longer than the other wild mountain gorillas in comparable “protective environments” where no significant postreproductive lifespan has been detected (6, 7). Therefore, “favorable” conditions, in both Ngogo chimpanzees and Bwindi gorillas, may in fact only offset historical human disturbances including hunting pressure, anthroponoses and habitat destruction (4), thereby revealing an adaptive trait that remains concealed in other extant populations which experience relevant disturbances.

Postreproductive lifespan may have not been recorded in other contemporary gorilla populations due to the difficulty of collecting decades-long data on wild gorillas and/or due to historical anthropogenic disturbance in gorilla natural environments. The combination of long-term studies of wild gorillas with the recent application of more favorable conditions described in the previous paragraph may allow us now to detect a trait that has been potentially common in the evolutionary history of gorillas. Be that as it may, our study adds to observations of postreproductive lifespan chimpanzees and humans, and refines our understanding on hominid life history evolution.

## Materials and Methods

We calculated i) survival probability to age *x*, using a Cox proportional hazards model which takes into account left-truncation (individuals entering the study later than birth) and right-censoring (individuals exiting the study before death; Fig. 1); ii) fecundity at age *x*, as the number of births observed at each female age divided by the number of females observed at that age; and iii) individual years lived after *x* (T*_x_*). We calculated PrR as the proportion of adult female years in the population lived by postreproductive females [years lived by females after 95% of population fecundity/years lived by females after 5% of population fecundity; $PrR=T_{95\%}/T_{5\%}$; (11)]. We also compared the calculated PrR value to a distribution of PrR values generated by randomly shuffling fecundity across ages for 10,000 iterations, to test whether it is significantly greater than zero. Finally, to assess the impact of age estimation errors on PrR values and significance, we repeated the calculation and permutation-based significance test 1,000 times: in each iteration, we randomly adjusted individual entry and exit ages by drawing an error term from a uniform distribution bounded by the estimated age error (min/max iteration age = estimated age ± age estimation error), we recalculated survivorship and fertility schedules, and recorded PrR and its *P*-value.

## Supplementary Material

Dataset S01 (CSV)

Code S01 (R)

## Data Availability

Data and code have been deposited in GitLab (https://gitlab.com/nksmt/post-reproductive-life-span-in-wild-mountain-gorillas) (10).
